# Foreign Bodies in the Air Passages

**Published:** 1907-11-16

**Authors:** 


### Diseases of Children.

### FOREIGN BODIES IN THE AIR PASSAGES.

The habit in children of putting objects into the mouth is a potent source of the passage of foreign bodies into the larynx, trachea and bronchi. In infants allowed to crawl along the floor a history is often unobtainable, and a mother will assure you positively that it is impossible such an accident can have happened. The actual cause of the inhalation is a sudden inspiration, such as may be started in older children by violent laughter or an unexpected slap on the back. Frequently the presence of a foreign body is not thought of until it is coughed up or found after death. The variety of objects is large, and includes peas, beans, husks of beech nut, nut-shells, pith of elder, buttons, beads, pebbles, fruit stones, coals, pins, penholders, pieces of pottery, glass stoppers, toy squeakers, etc.

The effects are immediate and remote. They vary with the size and nature of the object and the situation. The immediate effects are due to laryngeal irritation. Smooth, hard substances produce less immediate effect, but are more readily coughed up into the larynx, causing recurrent attacks of glottic spasm. The symptoms are often alarming. Dyspnœa is great; the child may become black in the face and die from suffocation. Usually the body passes back into the trachea and the symptoms subside, recurring again if, on coughing, the object impinges on the laryngeal structures. A small hard body remaining in the larynx causes paroxysmal cough, and perhaps hoarseness, dyspnœa and stridor. Paralysis of the laryngeal adductors has also been noted as an immediate effect.

Soft bodies and those which become soft from soaking in warm moist mucus are less likely to be coughed up. By their swelling they become firmly impacted and cause complete obstruction.

If the object remains in the trachea, it gives rise to respiratory obstruction, recession, stridor, and sometimes pain on swallowing. It usually passes onward into a bronchus, more commonly the right, and causes unilateral physical signs. In the early stages the movement of the chest is deficient; the breath sounds are weak or absent either over the lower lobe or the whole lung; resonance is impaired, the voice sounds weak, and there is unilateral recession. Sometimes stridulous wheezing on deep inspiration, paroxysmal cough, and pain in the side or epigastrium are present. Later on moist rales develop. The cough varies and may be absent, until septic broncho-pneumonia results from the retention of the secretions, local ulceration and microbial infection. The lung affection often ends in multiple abscesses, of varying size, sometimes subpleural; a variable degree of fibrosis; bronchiectasis; empyema; rarely gangrene. Compensatory emphysema may occur; so, too, may fatal hæmorrhage. At first the pulse is unduly frequent, respiration hurried, and there is a rise of temperature of about two degrees, for which it is difficult to account. Cough and expectoration come on within a week, and an appreciable amount of pus may be found in five or six days. The later symptoms are due to sepsis, and include hectic fever, malnutrition, troublesome cough, purulent and offensive sputa and diarrhœa. Cases often do not come under notice until local ulceration and suppuration have begun, because of the absence of early symptoms.

### Diagnosis and Treatment.

The diagnosis may be very difficult or equally easy. It is almost an axiom of medicine that in unilateral disease of the lung with purulent expectoration and signs of a cavity, the presence of a foreign body should be suspected. This applies with especial force to cases in which there is a past history of a choking attack, and an absence of the signs of empyema or tuberculosis. It is the association of the history of a choking fit, though even remote in point of time, with unilateral chest symptoms to which attention must be directed. The choking fit may not occur if the object is smooth and comes to rest at once. Recurrent attacks of dyspnœa are strongly confirmatory, if laryngeal mischief can be excluded. In one recorded case, a girl, aged nine, would hold the trachea tightly to prevent a stone being coughed up into the larynx and causing suffocating attacks. An *x*-ray examination may afford conclusive evidence.

Many cases get well in time by expulsion of the object or its encapsulation, but quite half die if left alone, from the secondary septic inflammation, sudden cardiac failure, or some complication. Bronchiectasis is a common sequel. Objects may be retained for many years.

*Treatment.*—Avoid the use of emetics, patting on the back and inversion of the body. Such measures are liable to cause the object to pass into the larynx and bring on fatal spasm. If adopted, everything must be ready for immediate tracheotomy. With this precaution, it is a justifiable mode of procedure if the patient is a girl of the higher social grade and the foreign body is a smooth one.

Tracheotomy is the proper treatment, for it at once reduces or stops the risks of glottic spasm, and affords a better outlet for the object. The trachea should be opened below the isthmus of the thyroid gland. The foreign body will often be at once coughed up through the wound, held widely open and aided by inversion. Failing this, an attempt must be made to locate it by a suitable probe, and to remove it by crocodile forceps, wire loop, or other suitable appliance. The tube can be taken out next day, unless there is a possibility that portions of a soft, friable object still remain.

In a few rare cases it may be advisable to attempt removal through the pleural cavity and the posterior mediastinum, or, in the case of the right bronchus, through the anterior mediastinum. These are very difficult and dangerous operations. Local suppuration and gangrene of the lung may necessitate local drainage.

